# Innovating the Instruction of Mathematical Concepts: How Does the Integrated Use of Digital Games and Language-Based Teaching Matter?

**DOI:** 10.3389/fpsyg.2022.858442

**Published:** 2022-04-22

**Authors:** Jiayao Shi

**Affiliations:** School of English and International Studies, Beijing Foreign Studies University, Beijing, China

**Keywords:** DGBL, mathematical concept teaching, systemic functional linguistics, k-12, integration of meaning and language

## Introduction

Digital game-based learning (DGBL) refers to the development and use of digital games (e.g., computer games) for educational purposes (Prensky, 2001). A DGBL activity is an activity engaging students in the process of problem solving or knowledge acquisition (Huang et al., 2010; Hwang et al., 2013).

Empirical studies have suggested that the use of digital games in mathematics education is an innovative teaching method that yields abundant benefits (see also Kirikkaya et al., 2010 for a review). First, DGBL increases students’ motivation and enthusiasm toward mathematics and helps transfer this positive attitude to learning both inside and outside the class (Becker, 2001; Cai et al., 2006; Ke and Grabowski, 2007; Ke, 2009). Second, DGBL enhances students’ knowledge building by facilitating teacher-student interaction or peer collaboration (e.g., teacher’s instant feedback, peer assistance in games), free from the constraint of time and place (White and McCoy, 2019). Third, DGBL enhances students’ learning by giving them a sense of control and achievement as they can progress through the game at their own pace, significantly raising their confidence in mathematics learning (Ku et al., 2014; Hulse et al., 2019). Overall, the use of digital games has been proved to be instrumental in teaching mathematics at the K-12 level based on its technological functions.

Nevertheless, DGBL of mathematical concepts needs to be further developed, as this technology-enhanced tool still cannot meet the complex demands of K-12 mathematics teaching. In particular, mathematics questions include the recounting of problem-solving procedures, describing a mathematical property, explaining a mathematical solution, or arguing a mathematical proof (Schleppegrell, 2004), all of which involve meaning making, and need teachers to help students understand the relationship between language (e.g., vocabulary and grammar) and meaning. However, many mathematics teachers are not familiar with the relationship between language and meaning (Accurso et al., 2017) and may feel puzzled about how to connect them when using DGBL. In addressing these problems, this paper recommends using SFL, a meaning-making approach which connects language and meaning, to guide the teaching of mathematical concepts in DGBL classroom. Deconstructing a mathematical question using the linguistic methods prescribed by SFL may help create effective DGBL tools which help divide the teaching of those concepts in manageable parts that combine to teach complex mathematical concepts. It hopes to provide recommended practices for K-12 mathematics teachers in English-speaking countries.

## Theoretical Framework: Understanding Mathematical Concepts Through Systemic Functional Linguistic

Systematic functional linguistics (SFL) is a suitable tool for complementing DGBL-based mathematics education because it explains how meanings are made in relation to language resources pertaining to a particular subject (e.g., mathematics) (Schleppegrell and Fang, 2008).

From the perspective of SFL, each discourse (e.g., a mathematical concept) has three levels of meaning, with each level of meaning involving a realization of a contextual variable at a higher level. The first one is the ideational meaning, which represents the general idea of a discourse (e.g., a mathematical question) and logical relations within the discourse (e.g., a causal relationship within a mathematical question). This level of meaning is a response to the contextual variable field, namely what a discourse is about. The interpersonal meaning is about the social relations conveyed in a discourse (e.g., an objective tone or subjective tone). This level of meaning involves a realization of the contextual variable tenor, namely the relationship between discourse participants (e.g., the writer of a math question, students who work on the math question). Finally, the textual meaning is concerned with the fluency or information flow of a discourse, which is a realization of the contextual variable mode (i.e., whether a mathematical question is delivered through a written or spoken mode). On all levels of meaning, SFL provides a transparent explanation on what meanings a discourse (e.g., a mathematical question or answer) is comprised of Schleppegrell (2001). The examples below show clearly how to understand certain mathematical concepts or mathematical problems from the perspective of each of the three levels of meanings. More importantly, what makes SFL useful is that it also provides labels (similar to the labels used for explaining the sentence structure in traditional grammar) and demystifies the connection between language resources (grammar and vocabulary) with the meanings aforementioned, explaining what specific linguistic resources can be used to construct or de-construct the meaning of a discourse. This means that students can be directed in a more specific way, using linguistic resources as a gateway to unpack/construct mathematical discourse.

In particular, the linguistic resources for constructing each of the ideational meanings are explained with the help of labels, such as participants (the label for nouns or noun phrases), process types (the label for verbs or verb phrases), and circumstances (the label for prepositional phrases), along with logical connectors (the label for logic relationships). Take the following mathematical concept as an example: “The mean score is the sum of the scores divided by the number of scores” (Veel, 1999, p. 196). The label participant can be used to identify the nouns or noun phrases (i.e., the mean score, the sum of the scores, and the number of scores) in the sentence, revealing the key constituents of the content as well as the features of nouns/noun phrases in relation to this mathematical topic. Unlike the everyday use of “mean,” “mean” as a technical lexical item in the labeled expression “the mean score” particularly merits attention, showing the mystery about this mathematical concept. Similar analysis through the label of participants could reveal the technical use of noun phrases like the sum of the scores and the number of scores in relation to this particular topic on the mathematical concept. The label process enables the identification of verbs or verb phrases, further showing the semantical relationship between participants aforementioned as well as the features of the verb/verb phrases. In the main clause, the word “is” shows the parallel relationship of the two participants (i.e., the mean score, the sum of the scores) while demonstrating itself as a “be” verb. The verb phrase divided by is also a process, showing the relationship between the sum of the scores and the number of scores, and is itself a verb phrase comprised of an action verb divide. These labels could help understand the meaning constituents at the ideational level, while revealing the features of language resources.

The labels provided by SFL for understanding interpersonal meaning include mood (the label for identifying whether a discourse is declarative, interrogative, or other) and appraisals (the label for identifying adjectives/adjective phrases, adverbs/adverb phrases, and modal verbs, or even other types of lexical categories/phrases that may convey evaluative stances). Take “The mean score is the sum of the scores divided by the number of scores” as an example (Veel, 1999, p. 196). With the label mood, it can be shown that the mathematical concept is declarative, showing that a mathematical concept is a way of declaring information. The linguistic features are the order of subject (the mean score) followed by the predicate (is). With the label appraisal, it can be found that the evaluative stance is objective, as also illustrated by the use of non-human nouns and non-evaluative linguistic resources. In other words, a mathematical concept, at the interpersonal level, is declarative, with an objective tone, which is realized through corresponding linguistics resources.

Textual meaning can be approached through labels at two levels. One is cohesive devices functioning between two or more than two sentences. They are used for identifying the use of grammatical resources (i.e., conjunction words) or lexical resources, including but not limited to pronoun reference, the repetitive use of the same word, antonyms, or synonyms. At the other level are theme and rheme, which identify how information flow is organized within a sentence or in relation to other sentences. The label theme is used to identify elements that start a sentence. The rest of elements within a sentence are labeled as rheme. Again, take the concept of the mean score The mean score is the sum of the scores divided by the number of scores as an example. Since the concept is represented through one sentence, the label theme and rheme can be used to illustrate the construction of its textual meaning. The theme of the sentence is The mean score, and the verb phrase (with be as the verb) is the rheme. The way the definition is conveyed through a term to be explained, followed by a verb phrase, instead of the other way round, reflects how information in a definition is organized. Using the labels of theme and rheme can also identify the features of its mathematical formula. The corresponding formula for the aforementioned concept is “ $\bar{X}=\sum x/n{}^{\prime \prime }$(Veel, 1999, p. 196). In the formula, $\bar{X}$, which stands for the mean score, is used to visually start the formula, while the symbol = Σx/n, as the rheme of the whole formula, is used to visually represent the rest of information in the definition. In sum, the labels of theme and rheme reflect how the definition is visually and sequentially represented.

Based on the above, integrating DGBL with SFL would be a promising way to offer students an effective way of learning mathematical concepts, with the focus on language. Research is abundant on both SFL’s usefulness and its empirical use in non-digital game-based mathematics classrooms (Zolkower and Shreyar, 2007; Shreyar et al., 2009; Accurso et al., 2017) and the use of DGBL in mathematics classrooms (McCoy, 1996; Callaghan et al., 2018; Noah, 2019). It can be assumed that the integration of the technological uses of DGBL with SFL can further improve the teaching of mathematical concepts. However, few classrooms have integrated the two constructs, which may be explained by the limited promotion of these two integrated constructs among mathematics teachers. In order to fill this gap, this paper discusses how to prepare K-12 mathematics teachers for understanding and harnessing these two constructs. It is hoped that through the suggestions provided, the teaching of mathematics in English-speaking countries could be made both interesting and explicit.

## Pedagogical Design of Mathematical Concepts Teaching: Integrating Digital Games and SFL-Informed Teaching

This section provides some suggestions as to how mathematics teachers can leverage affordances of both DGBL-based mathematical concepts teaching and SFL. The combined affordances include supporting students in deconstructing mathematical concepts through SFL-informed teaching (Schleppegrell, 2007; Shreyar et al., 2009; O’halloran, 2011; Zolkower and de Freitas, 2012), while creating engaging and motivating contexts through DGBL (Mokka et al., 2003; Natvig and Line, 2004; Ebner and Holzinger, 2007; Ke, 2009).

### Creating Context Through Digital Game-Based Learning

When teaching mathematical concepts, digital games are used to motivate students and create a positive attitude toward mathematics. That is, the first step is to create an engaging context that relates to a mathematics concept through DGBL. For example, in a digital game focused on the mean score, students are expected to engage in the activities to be prepared for the follow-up learning and application.

Take the “Balloon Shooting” activity as an example (Hwa, 2018). In this game, students will find that on the left side of the screen, there are several balloons, each with a number on it and on the right side is an animated cat. The cat keeps asking for help with questions related to the mean score, like “I have 21 fish, and if I want to equally divide them and have the same number of fish for a whole week, how many should I eat every day?” and “Luna wants to make some cookies and to share them with her classmates. If she has 20 cookies in total and there are 10 students in her class, how many cookies will each of her classmates get?” In response to the cat’s questions, students need to move the shooting cursor of their computer mouse to shoot the correct answer (the number on the balloon). If the answer is right, the animated cat will smile and praise the student with a “Well done.” If it is wrong, the cat will put up a new balloon and encourage the student to “Try again.” In this gaming environment, students will be motivated and willing to engage in the problem-solving process.

Each game provides the student with levels of increasing difficulty. As students complete each level, they progress to a new and slightly more difficult level, such as more questions or extension to larger numbers. Consequently, with practice, students can gain a basic understanding about the concept of the mean score (e.g., the mean score is equal to the number of cookies everyone gets in the class) as well as being motivated to learn more about the mean score so that they can reach the next level and be leading in the game.

During the game, they will also encounter questions related to the definition of the topic and its possible use in real-life situations, which helps them better contextualize the concepts. For example, they may be confused about what the meaning of each part in the formula is. Questions such as “What is the relationship between “X” and “x” in the formula?” and “Can you explain the definition of the mean score to your classmates?” can DGBL create an engaging venue to help students became familiar with the background needed for understanding follow-up deconstruction of mathematical concepts.

The second step is implementing SFL-informed teaching through the means of DGBL. That is, students are taught about the relation between language resources and content through DGBL as a tool. Informed by the construct of ideational meaning and the contextual variable field, when K-12 teachers try to engage their students in understanding the meaning of certain mathematical concepts, they can remind their students, in plain language rather than technical terms, to focus on how the ideational meaning of a concept is realized through topic-related linguistic resources (Rose and Martin, 2012). This can be achieved effectively through DGBL, for instance, by helping students identify the type of noun or noun phrase (*participants*, technical words in particular) and the verb or verb phrase (*processes*) in use (Rose and Martin, 2012). Again, take teaching the concept of the mean score as an example. In the “Card Matching” game (Hwa, 2018), students’ understanding of nouns, noun phrases, verbs, and verb phrases can be tested. That is, at the bottom of the screen, all types of participants (nouns and noun phrases)—namely, actor, agent, theme, patient, experiencer, goal, recipient, location, and instrument—will be presented in the form of a card. The definition of the mean score, “*The mean score is the sum of the scores divided by the number of scores*,” will appear at the top of the screen. Under each word, there will be a blank card, and there will be confusing options. In this game, students are expected to drag the cards at the bottom with their mouse and to match them with the blank cards below the definition. Only when students match all words correctly can they move on to the next level.

In terms of interpersonal dimension, for the concept of the mean score, teachers can help students realize the declarative mood and objective tone used in the presentation of this mathematical concept by using the “Comparison” game. During this game, several forms of mood and tone will be employed to represent the concept at the same time. Faced with various options on their screen, students are expected to compare these moods and tones and choose the most appropriate one. Only when the declarative mood and objective tone are chosen can they win the game. In this way, students will know that mathematical concepts should be presented in a declarative mood and objective tone. Meanwhile, teachers could also use simple and accessible language to explain this to enhance students’ understanding of the interpersonal dimension of the mathematical concept.

Teachers can also help their students understand the interaction between language resources and textual meaning embedded in mathematical concepts through DGBL. For instance, understanding of cohesive devices can be cultivated from the “Draw the Picture” game (Hwa, 2018). Each click on a cohesive device used in a paragraph will make it turn into one piece of a picture. A complete picture will appear after students point out all the cohesive devices. Students may recognize some devices and infer others, which will build their understanding. In terms of theme and rheme, “Draw the Picture” can also be used but with theme and rheme as the targets for recognition. Again, students’ participation in the game can be further supported by their teachers’ explanation of textual meaning and its linguistic realization through plain language. In this way students may understand textual resources in discourses through self-exploration in digital games.

With the help of SFL in digital games, students are able not only to deconstruct mathematical concepts but also to stay motivated during the whole process. Immersed in a motivating digital game environment and supported with SFL, students may develop a better understanding of mathematical concepts.

### Teachers’ Efforts in Implementing Integrated Teaching

K-12 level teachers also need to be able to implement the aforementioned integrated framework flexibly. It is expected that teachers assist students in diverse ways to meet their varied needs (Leikin and Dinur, 2007; Valoyes-Chávez, 2019). In other words, besides following the framework provided above, K-12 mathematics teachers also need to get ready to make adaptions according to their students’ needs through an ongoing process of self-reflection on their practice (Cross, 2009).

Teachers can achieve this by analyzing students’ performance (Fan, 2011), either in class or on the online digital game platform. For example, by observing students’ performance in class (e.g., whether students interact with the teacher and nod to show their understanding) and on the online digital-game platform (e.g., students’ operation will be recorded by the computer so that teachers can see whether they have made the right choice based on their understanding of the concept or through a lot of trial and error), teachers can gain a more comprehensive understanding of the extent to which the whole class has grasped certain mathematical concepts and make subsequent adjustments to the teaching plan if needed. Students’ performance on exams are also a form of direct feedback, which helps to inform adjustments in teaching. Additionally, teachers can evaluate students’ responses by listening to their opinions (Canning, 2017; Deng et al., 2020) on matters such as how they perceive the course and what their expectations from the course are. In this way, teachers can get first-hand feedback on instruction, such as whether students find this new teaching framework useful. This direct student feedback can be achieved through multiple channels, such as interviews, written reflections, or surveys (Johnson and Christensen, 2012). By critically evaluating students’ reactions, teachers can adjust their teaching accordingly.

Adapting to such a new integrated framework may require additional teaching and learning, which may pose certain emotional challenges for both the teacher and the students (Cross, 2009; Bell and Gresalfi, 2017; Deng et al., 2020). In this case, adequate attention should be paid to their emotions. Teachers need to gradually improve students’ performance and boost their confidence (Lei et al., 2018), especially those who do not do well in mathematics and digital games, in case they are frustrated with a new way of teaching. What’s more, teachers will also need to do a lot to implement the new framework to meet students’ diverse needs. They should also be provided with emotional and technical support (Deng et al., 2020). For example, they can work with their colleagues for ideas on how to better integrate the above framework into their teaching and share with them the difficulties faced in its implementation.

## Conclusion and Further Research

This paper discusses the potential of integrating SFL with DGBL in K-12 mathematics teaching. In particular, it highlights the important role SFL plays in enhancing DGBL of mathematical concepts by revealing the connection between language and content. Sample teaching practices are also provided for teachers on how to integrate DGBL of mathematical concepts and SFL and how they can better apply this integrated framework in the teaching of mathematical concepts.

This research has some limitations. First, this study only discusses the possibility of such an integrated framework at the theoretical level. Second, this paper does not go into detail about how to integrate SFL with DGBL in teacher-training programs.

Future research is warranted in the following aspects. First, empirical research could be conducted to investigate the impact of the integrated use of SFL and DGBL-based mathematical concept teaching on K-12 students. Second, studies could also explore whether and how teachers’ pre- and in-service training programs integrate SFL-based instruction with DGBL. Third, this study only discusses the possibility in K-12 education, so research on whether this framework can be applied in other contexts, such as with English as a Second Language students at the tertiary level or other contexts where students have similar challenges with the learning of mathematical concepts, would be of interest.

## Author Contributions

The author confirms being the sole contributor of this work and has approved it for publication.

## Conflict of Interest

The author declares that the research was conducted in the absence of any commercial or financial relationships that could be construed as a potential conflict of interest.

## Publisher’s Note

All claims expressed in this article are solely those of the authors and do not necessarily represent those of their affiliated organizations, or those of the publisher, the editors and the reviewers. Any product that may be evaluated in this article, or claim that may be made by its manufacturer, is not guaranteed or endorsed by the publisher.
